# Contribution of Connexin Hemichannels to the Pathogenesis of Acute Lung Injury

**DOI:** 10.1155/2020/8094347

**Published:** 2020-11-17

**Authors:** Shuaiwei Wang, Yafang Sun, Yu Bai, Nannan Zhou, Na Chen, Edmund J. Miller, Yijie Zhang, Wei Li

**Affiliations:** ^1^Sepsis Laboratory, Center for Translational Medicine, Huaihe Hospital, Henan University, Kaifeng, Henan, China; ^2^International Laboratory for Sepsis Research, Kaifeng, Henan, China; ^3^Engineering and Research Center for Sepsis, Kaifeng, Henan, China; ^4^Department of Pulmonary and Critical Care Medicine, Huaihe Hospital, Henan University, Kaifeng, Henan, China; ^5^RDS2 Solutions, 25 Health Sciences Drive, Suite 208-B, Stony Brook, NY 11794, USA

## Abstract

Connexin (Cx) family members form hemichannels (HCs) and gap junctions (GJs). Biological functions of Cx HCs have not been adequately characterized due to the inability to selectively target HCs or GJs. Recently, we developed a 6-mer peptide mimetic (P5) of the first extracellular loop of Cx43 and showed that it can block the permeability of HCs but not GJs formed by Cx43. In this study, we further characterized the HC blocking property of P5 and investigated the role of Cx HCs in acute lung injury (ALI). We found that P5 administration decreased HC permeability, in pulmonary microvascular endothelial cells, HepG2 cells, and even Cx43-deficient astrocytes, which express different sets of Cxs, suggesting that P5 is a broad spectrum Cx HC blocker. In addition, P5 reduced HC permeability of alveolar cells *in vivo*. Moreover, P5 decreased endotoxin-induced release, by vascular endothelial cells *in vitro*, of high mobility group box protein 1 (HMGB1), a critical mediator of acute lung injury (ALI), and reduced HMGB1 accumulation in bronchoalveolar lavage fluid (BALF) of mice subjected to intratracheal endotoxin instillation. Furthermore, P5 administration resulted in a significant decrease in the concentrations of ALT, AST, and LDH in the BALF, the accumulation of leukocytes in alveoli, and the mortality rate of mice subjected to ALI. Wright-Giemsa staining showed that P5 caused similar reductions of both neutrophils and monocytes in BALF of ALI mice. Together, these results suggest that Cx HCs mediate HMGB1 release, augment leukocyte recruitment, and contribute to ALI pathology.

## 1. Introduction

Acute respiratory distress syndrome (ARDS) is an intense inflammatory disorder, characterized by severe respiratory failure requiring mechanical ventilation, that occurs within a week after initial pulmonary or nonpulmonary insults [1]. Despite progress in diagnosis and treatment, the development of novel therapies in ARDS has been extremely challenging and the mortality rate of ARDS remains as high as 46% among the 3 million patients diagnosed annually worldwide [2, 3]. The prognosis of ARDS is dependent on the severity of the lung injury [2, 3].

ARDS-associated acute lung injury (ALI) is characterized by widespread inflammation and subsequent alveolar epithelial-endothelial barrier damage, which are largely attributable to a variety of inflammatory mediators including TNF-*α*, IL-8, IFN-*γ*, and HMGB1 [2–5]. Extracellular HMGB1 has been shown to be critical for ALI associated with both sterile (e.g., trauma) and nonsterile (e.g., sepsis) insults [5, 6]. Under physiological conditions, HMGB1 is predominantly located in cell nuclei but can be actively released by activated innate immune cells, such as macrophages and neutrophils, during inflammation [7, 8]. In contrast to TNF-*α* and IFN-*γ*, which are released by macrophages within 2 hr after endotoxin challenge, a significant release of HMGB1 occurs much later [7, 8]. Moreover, extracellular HMGB1 can stimulate immune cells to release early cytokines [9]. These attributes render HMGB1 a late mediator of inflammation and hence an important target for potential therapies of inflammatory diseases such as ARDS and sepsis [7–9].

Several lines of study suggest that connexin (Cx) channels participate in the regulation of inflammatory responses in ALI [10–21]. Indeed, a blockade of Cx channels reduces the accumulation of HMGB1 in the culture medium of LPS-challenged macrophages and in the serum of septic mice [10, 11]. Moreover, changes in Cx protein level or Cx channel permeability correspond to alterations in inflammation, alveolar barrier integrity, and mortality rates, demonstrating the significance of Cx channels in the pathogenesis of ALI [12–21]. However, it is not known if Cx channels are involved in the regulation of HMGB1 release in ALI.

It is well established that Cx family members form two types of membrane channels: hexameric hemichannels (HCs) and gap junctions (GJs) assembled by two apposing HCs. Thus, Cx HCs and GJs have identical size and constituent structural proteins and thereby have similar physical properties such as permeating crosschannel flow of molecules under 1.2 kDa [22, 23]. HCs mediate communication between the cell interior and extracellular milieu whereas GJs mediate intercellular communication, suggesting that these channels serve different biological functions. However, defining the functional distinctions between HCs and GJs has proven challenging due to the lack of means to selectively target either channel type [22, 23].

We recently screened a panel of Cx43 mimetic peptides and found that a 6-mer peptide, termed P5, can selectively block HCs but not GJs in Cx43 expressing macrophages and NIH3T3 fibroblasts. Moreover, P5 can dose-dependently inhibit HMGB1 release by macrophages and reduce the mortality rate in a mouse model of sepsis. Furthermore, P5 can attenuate ischemic liver injury associated with ischemia/reperfusion [10]. Since the amino acid sequence of P5 is shared by several other members of the Cx superfamily (Table 1), we hypothesize that it may block HCs formed by different Cxs. In this study, we further characterized the Cx HC blocking property of P5 and investigated the role of Cx HCs in the pathogenesis of ALI.

## 2. Materials and Methods

### 2.1. Materials

Bacterial endotoxin (lipopolysaccharide, LPS, *E. coli 0111:B4*) and Lucifer yellow (LY, L0144) were obtained from Sigma-Aldrich (St. Louis, MO, USA). Paraformaldehyde (PF) was from Damao Chemical (30525894, Tianjin, China). Rabbit antibodies against HMGB1 (10829-1-AP), Cx43 (26980-1-AP), alanine aminotransferase (ALT, 16897-1-AP), aspartate aminotransferase (AST, 14886-1-AP), and lactate dehydrogenase (LDH, 19987-1-AP) were from Proteintech (Rosemont, IL, USA). Rabbit anti-Cx37 (ab181701) and Cx40 (ab38580) antibodies were from Abcam (Cambridge, MA, USA). HRP-conjugated donkey anti-rabbit IgG was from GE Healthcare (NA934, Port Washington, NY, USA). Cx mimetic peptide P5 (ENVCYD) was synthesized, purified, and salt exchanged by GenScript (purity > 95%, Nanjing, China). Fetal bovine serum (35-081-CV) and Dulbecco's modified Eagle medium (10-013-CVRC) were from Corning (Corning, NY, USA). Antibiotics (100x penicillin and streptomycin solution) were from Gibco (15140-122, USA). DAPI was from Solarbio (D8200, Beijing, China). Mitomycin C was from TCI (M2320, Shanghai, China). PVDF membrane was from Bio-Rad (1620177, Hercules, USA). Wright-Giemsa staining kit was from BASO Biotech. (BA-4017, Zhuhai, China). Macrophage cell line RAW264.7, hepatocyte cell line HepG2, NIH3T3 fibroblasts, and the human pulmonary microvascular endothelial cell line (HPMEC-ST1.6R) were kindly provided by C. James Kirkpatrick (Institute of Pathology, Johannes-Gutenberg University, Germany). Cx43-deficient mouse cortical astrocytes were generous gifts from Dr. David C. Spray (Albert Einstein College of Medicine, NY).

### 2.2. Cell Culture

RAW264.7 cells, NIH3T3 fibroblasts, HepG2 cells, HPMVEC, and Cx43-deficient astrocytes were cultured in DMEM supplemented with 1% penicillin/streptomycin and 10% fetal bovine serum, as described previously [10, 11]. For experiments involving different treatments, equal numbers of cells were seeded in each well of multiwell culture plates. When reaching 70–80% confluence, cells were washed with, and cultured in, serum-free DMEM before the administration of LPS (0.5 *μ*g/ml), in the absence or presence of P5 (10-20 *μ*g/ml). Extracellular release of HMGB1 by cultured HPMVEC and the expression of Cx43 by NIH3T3 and HepG2 cells were determined by Western blotting.

### 2.3. HC Permeability Assays

LY dye uptake assay was used to examine the effects of P5 on HC permeability, as previously described [10, 11]. Briefly, HPMVEC, HepG2 cells, and Cx43-deficient astrocytes were subjected to LPS (0.5 *μ*g/ml) stimulation for 16 hr before the administration of P5 (20 *μ*g/ml). It should be noted that these treatments did not cause apparent cell death in these assays (not shown) and thus should not activate pannexin 1 channels [24]. Five minutes later, calcium chelator EDTA (1 mM) and LY (0.1%) were sequentially added to the culture medium and incubated for 10 min. Cells were then fixed with 2% PF and stained with DAPI. LY and DAPI signals were acquired using an inverted fluorescent microscope equipped with a CCD camera (Eclipse Ti2, Nikon, Japan). ImageJ software was used to threshold and then quantify LY and DAPI signals. DAPI value reflects total cell counts, whereas LY value reflects total hemichannel capacity, and hence, the ratio of LY and DAPI was used as the measurement of HC permeability, as previously described [10, 11].

To determine the effects of P5 on HC permeability *in vivo*, LPS was administered intraperitoneally (5 mg/kg, 14 mice per group) to induce uniform pulmonary inflammation [25]. After 20 hr, P5 (8 mg/kg) and LY (10 mg/kg) were sequentially administered. Mice were transcardially perfused 10 min later from the right ventricle towards the left atrium with 0.1 M phosphate-buffered saline and 2% PF. The lungs were then removed, sectioned (10 *μ*m) using a cryostat (Leica CM 1950, Germany), and stained with DAPI. The fluorescent signal of LY and DAPI was acquired, and HC permeability was determined, as aforementioned.

### 2.4. Animal Model of ALI

A total of 218 Balb/c male mice (9-10 wks old, 23-27 g) were purchased from Model Animal Research Center of Nanjing University (Nanjing, China), maintained at the Center for Translational Medicine at the Huaihe Hospital of Henan University, and used for this study in accordance with the guidelines of the Animal Care and Use Committee of the Huaihe Hospital of the Henan University (Animal Protocol # 2017031).

Animals were divided into 4 groups: Sham (surgery+saline), P5 (surgery + 8mg/kg P5), ALI (5 mg/kg LPS+saline), and ALI+P5 (5 mg/kg LPS + 8mg/kg P5), unless otherwise specified. ALI was induced by intratracheal LPS (5 mg/kg) instillation while the animals were under ketamine and xylazine anesthesia, as previously described [18, 21], and saline or P5 was administered (200 *μ*l, ip) twice at 2 and 16 hr postsurgery unless otherwise noted.

For survival studies, thirty mice were used for each of the ALI and ALI+P5 groups. The condition of each animal was checked thrice daily for 10 days following induction of ALI, and the numbers of surviving animals were recorded. For pathological studies, ten mice per group were used. At 4 hr after the second injection of saline or P5, mice were euthanized by CO_2_ asphyxiation, and lungs were fixed in situ and postfixed with 2% PF according to a previously described protocol [21]. Frozen sections (10 *μ*m) were subjected to H&E staining. Images of the entire sections were taken, and alveoli with or without leukocytes were counted in a double-blind manner. To determine extracellular levels of HMGB1, ALT, AST, and LDH, ten mice per group were used to harvest bronchoalveolar lavage fluid (BALF, 2 ml/mouse) at 4 hr postsecond administration of saline or P5, as previously described [15, 18]. The lung was subsequently removed for Western blotting analysis of Cx37, Cx40, and Cx43. For characterization of leukocytes in BALF, 10 mice per group were used. Half of the BALF was used to obtain total cell counts using a Sysmex Multispecies XN-10 flow cytometry system (Body Fluid mode, Lincolnshire, IL, USA). The other half was centrifuged at 1,000 g to obtain cell pellets. The cells were resuspended in 50 *μ*l mouse serum, smeared onto a glass slide, and subjected to Wright-Giemsa staining according to the manufacturer's instruction. Cell types were viewed and determined with a bright field microscope.

### 2.5. Western Blotting

The protein levels of Cx37, Cx40, and Cx43 in lung tissues; HMGB1 in culture medium; and ALT, AST, and LDH in BALF were examined by Western blotting, as previously described [10, 11]. Culture medium and BALF samples were concentrated into 100 *μ*l volumes by ultrafiltration (10 kDa cutoff, UFC801096, Millipore, USA), and 20 *μ*l of the resulting concentrate was used for Western blotting evaluation of each protein. For Cx expression, mouse lungs were homogenized using an ultrasonic tissue homogenizer (JY92-IIN, Scientz, Ningbo, China), and protein concentrations were determined using a BCA kit (AR0146, Boster, Wuhan, China). Tissue lysates containing equal amounts of protein were used for Western blot analysis. Protein signals after ECL (CW0049M, CWBio, Beijing, China) incubation were acquired using a ChemiDoc XRS system (Bio-Rad, Hercules, U.S.A.) and quantified using ImageJ software. Protein loading was quantified by densitometry of protein staining (GelCode blue, Cat# 1860983, Thermo, USA) and used to normalize Western blots.

### 2.6. Statistical Analysis

Values are expressed as means ± SE of at least three independent experiments. Student's *t*-test was used for comparison between two groups. One-way analyses of variance (ANOVA) followed by the Tukey test for multiple comparisons were used to compare between different groups. A comparison of Kaplan-Meier survival curves was conducted using the log-rank method. A *P* value < 0.05 of the two-tailed test was considered statistically significant.

## 3. Results

### 3.1. Cx43 Mimetic Peptide P5 Decreased the Permeability of HCs Formed by Different Cxs

We have shown previously that P5 can reduce HC permeability in Cx43 expressing macrophages. Furthermore, P5 does not interrupt Cx43 phosphorylation, GJ formation, or gap junctional intercellular coupling in NIH3T3 fibroblasts [10]. Since the entire amino acid sequence of P5 (ENVCYD) is also present in Cx33, Cx45, Cx46, and Cx50, in addition to Cx43, and the majority of P5 sequence (5 out of 6 amino acids, NVCYD) can be found in the same topological region (the first extracellular loop, EL1) in human Cx26, Cx30, Cx30.3, Cx31, Cx37, Cx40, Cx47, and Cx59 (Table 1), it is possible that P5 may also block HCs formed by Cxs besides Cx43 in different cell types.

**Figure 1:**
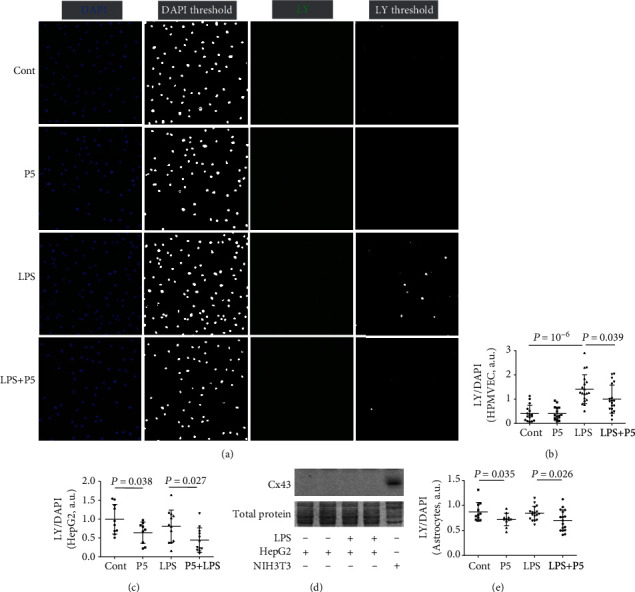
Cx43 mimetic peptide P5 inhibited LY uptake *in vitro*. HPMVEC (a, b), HepG2 (c), and Cx43-deficient cell (e) cultures were divided into Cont (saline), P5 (20 *μ*g/ml), LPS (0.5 *μ*g/ml), and LPS+P5 (0.5 *μ*g/ml LPS+20 *μ*g/ml P5) groups. LY was applied to the culture medium for 10 min incubation. Then, cells were fixed with paraformaldehyde and stained with DAPI. The ratio of LY/DAPI fluorescence signals was used to determine HC permeability. The unit of the LY/DAPI ratio is arbitrary, and the values are not comparable between experiments using different cells. (d) Expression of Cx43 in HepG2 and NIH3T3 cells with or without LPS challenge was examined using Western blotting. a.u.: arbitrary unit.

Therefore, in our initial studies, we tested whether P5 could block HCs in vascular endothelial cells that express Cx37, Cx40, and Cx43 [14, 16]. As shown in Figure 1(a), normal HPMVEC (Cont) exhibited a relatively low level of signal ratio of HC permeable tracer LY and cell nuclei marker DAPI (LY/DAPI). After 16 h, LPS had induced a near twofold increase (185%) of the LY/DAPI ratio, suggesting that LPS challenge caused a dramatic increase of HC permeability in HPMVEC. However, coapplication of P5 reduced the LY/DAPI ratio in LPS-treated cells by 30.4% (Figure 1(a)), demonstrating that P5 can also block HCs in HPMVEC, in addition to macrophages [10].

Because endothelial cells also express Cx43 [14, 16], we then determined whether P5 can inhibit the permeability of HCs formed by other Cxs. Control HepG2 cells exhibited a low-level hemichannel activity, reflecting perhaps a basal level of permeability under the experimental condition (Figure 1(c)). In contrast to inflammatory cells such as macrophages and endothelial cells, LPS treatment did not cause any changes in LY/DAPI ratio in HepG2 cells. However, P5 significantly (*P* < 0.05) reduced LY/DAPI ratio in control and LPS-treated cells by 27% and 28%, respectively (Figure 1(c)). Consistent with previous observations [26], no Cx43 was detected in HepG2 cell lysates even though it was abundantly present in NIH3T3 fibroblasts (Figure 1(d)). These results suggest that P5 blocks HCs formed by Cxs other than Cx43.

To confirm this notion, we performed a similar experiment using Cx43-deficient mouse cortical astrocytes (Figure 1(e)). Like HepG2 cells, a 16 hr treatment with LPS failed to induce any significant changes in the level of LY/DAPI ratio in astrocytes. However, a mild but significant reduction was observed in both Cont (12.14%) and LPS-treated cells (13.5%), demonstrating that P5 is a pan-Cx HC inhibitor that can block HCs formed by different Cxs.

### 3.2. P5 Reduced HC Permeability of Alveolar Cells In Vivo

**Figure 2:**
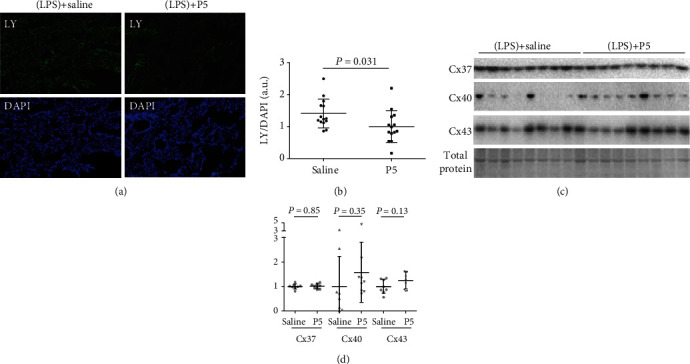
The effects of P5 on HC permeability (a, b) and Cx expression (c, d) of alveolar cells in vivo. (a, b) At 20 hr after LPS challenge, saline or P5 was administered (ip) twice at 2 and 16 hr post-ALI induction. After 10 min, mouse lungs were perfused with PBS, fixed with PF, and then cut into sections that were subsequently stained with DAPI. LY-containing cells appeared in green, showing LY uptake through HCs. Nuclei of all alveolar cells were stained with DAPI and appeared in blue. The ratio of LY/DAPI fluorescence signals was used to determine HC activity. a.u.: arbitrary unit. (c, d) Saline or P5 was administered (ip) twice at 2 and 16 hr post-ALI induction. After BALF extraction, lungs were harvested and homogenated to determine the expression of Cx37, Cx40, and Cx43 by Western blotting.

We next determined whether P5 can inhibit HC permeability *in vivo*. As shown in Figure 2(a), P5 reduced the intensity of the LY signal in the lungs of LPS-challenged mice, compared with saline controls. Consistent with pan-Cx HC inhibiting property, it appeared that the reduction of LY signal occurred in virtually all cell types in alveoli and their vicinity, which also suggests little, if any, off-target effect of P5 on pannexin 1 channels that are only open during apoptosis [24]. Quantification of the LY/DAPI ratio showed a reduction of 34.7% as a result of P5 administration (Figure 2(b)).

There are primarily two contributing factors of HC activity: the total number of channels and channel permeability. The former is largely determined by the levels of constituent Cxs. In particular, Cx37, Cx40, and Cx43 are major Cx isoforms expressed in the lungs [14–16, 20]. As shown in Figure 2(c), the expression of Cx40 exhibited a higher level of variability than Cx37 and Cx43 in ALI mouse lungs. P5 did not have any visible impact on the expression of Cx37 (*P* = 0.85) and Cx43 (*P* = 0.13) but was apparently associated with an elevation in Cx40 expression (Figure 2(c)). However, densitometry analysis did not reveal any significant differences in Cx40 between the P5 and the saline control (*P* = 0.35, Figure 2(d)). These results indicate that P5-induced reduction of the LY/DAPI ratio in alveolar cells of LPS-treated mice was unlikely due to reduce channel capacity but alternatively attributable to decreased permeability.

### 3.3. P5 Inhibited Extracellular Release of HMGB1 In Vitro and In Vivo

**Figure 3:**
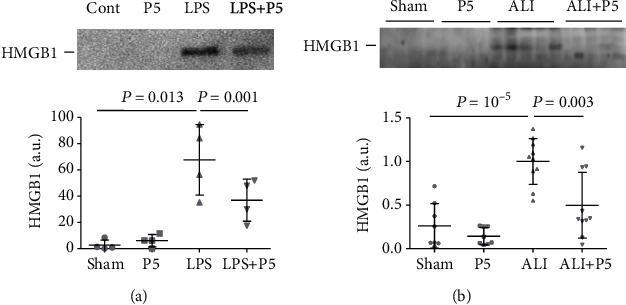
P5 inhibited the extracellular release of HMGB1 *in vitro* and *in vivo*. (a) Cultured HPMVEC were divided into Cont, P5 (20 *μ*g/ml), LPS (0.5 *μ*g/ml), and LPS+P5 (0.5 *μ*g/ml LPS and 20 *μ*g/ml P5) groups. At 16 hr post-LPS challenge, culture medium was collected, concentrated, and subjected to Western blotting analysis of HMGB1. *n* = 4. (b) Balb/c mice were divided into 4 groups: Sham (surgery+saline), P5 (8 mg/kg P5), ALI (5 mg/kg LPS+saline), and ALI+P5 (5 mg/kg LPS + 8mg/kg P5). ALI was induced by intratracheal instillation of LPS. Saline or P5 was administered twice through ip at 2 and 16 hr post-ALI induction. BALF was collected at 4 hr after second saline or P5 administration, concentrated, and subjected to Western blotting analysis of HMGB1. a.u.: arbitrary unit; *n* = 10.

We have previously observed that the blockade of Cx HCs by P5 resulted in the reduction of HMGB1 release by macrophages [10]. In light of HC inhibitory effects of P5 on HPMVEC, we examined whether P5 can also suppress HMGB1 release by these endothelial cells. Like macrophages, HPMVEC did not secrete a detectable amount of HMGB1 under physiological conditions (Cont in Figure 3(a)) but responded to LPS stimulation with a substantial release of HMGB1 (LPS in Figure 3(a)). Coadministration of P5 (LPS+P5) approximately halved (52.05%) the extracellular concentration of HMGB1 when compared with the LPS group.

We next determined whether the blockade of Cx HCs could also cause a reduction of extracellular HMGB1 in injured lungs. As shown in Figure 3(b), ALI caused a marked increase in the concentration of HMGB1 in BALF, which was reduced by 39.35% by P5. Taken together with the effects of P5 on HMGB1 accumulation by macrophages and endothelial cells, these observations support the hypothesis that HMGB1 release is controlled, at least in part, by Cx HCs in various cell types, both *in vitro* and *in vivo*.

### 3.4. HC Blockade Attenuated LPS-Induced Lung Injury

**Figure 4:**
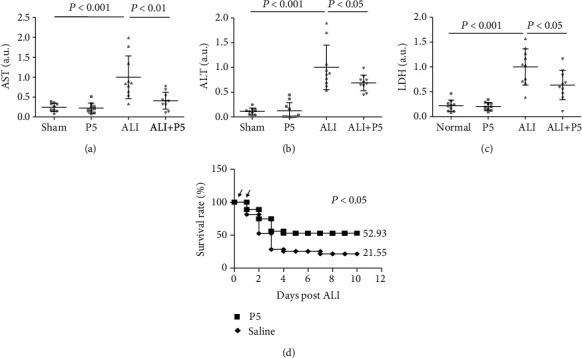
Effects of P5 on the levels of ALT (a), AST (b), and LDH (c) in BALF and survival rate (d) of ALI mice. (a–c) Mouse ALI induction and treatments were performed in the same fashion as in Figure 3(b). The relative levels of AST (a), ALT (b), and LDH (c) in BALF were assessed by Western blotting. *n* = 10. ^∗^*P* < 0.05. Values are in arbitrary units (a.u.). (d) Same volume (200 *μ*l) saline or P5 (8 mg/kg) was administered twice through ip at 2 and 16 hr post-ALI induction (5 mg/kg LPS, intratracheal instillation) in Balb/c mice. Survivors were counted daily for 10 days. Arrows indicate the time of saline or P5 injections, *n* = 30.

As extracellular HMGB1 may play a critical pathogenic role in ALI [5, 6], a reduction of HMGB1 release resulting from the HC blockade may have protective effects in ALI. Intratracheal instillation of LPS caused 7.95-, 3.08-, and 5.71-fold increases in tissue injury markers such as ALT, AST, and LDH in BALF 20 hr later, respectively (Figures 4(a)–4(c)). The increase in ALT may indicate a collateral liver injury resulting from LPS, dispersing from the alveoli into the circulation. The administration of P5 (twice at 2 and 16 hr post-ALI induction) resulted in 31.82%, 58.81%, and 36.85% decreases in ALT, AST, and LDH in BALF, respectively (Figures 4(a)–4(c)). More importantly, P5 administration significantly increased the survival rate from 21.55% to 52.93% (Figure 4(d)). These results suggest that the inhibition of HCs is protective against LPS-induced lung injury.

**Figure 5:**
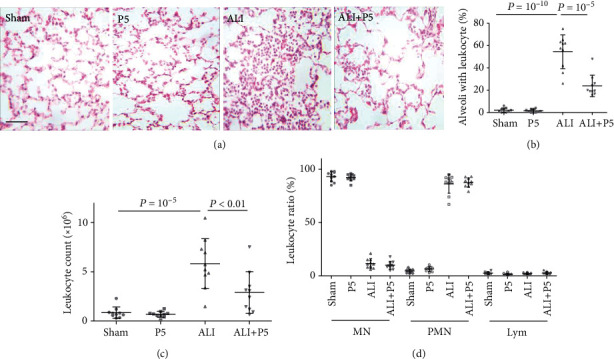
P5 reduced alveolar leukocyte recruitment. (a, b) Saline (Sham) or P5 (8 mg/kg, P5) was injected twice though ip at 2 and 16 hr post-ALI induction in mice. At 4 hr after second administration, mouse lungs were fixed in situ with PF, cut into 10 *μ*m sections, and stained with H&E. The counts of alveoli with and without leukocytes were obtained in a double-blind manner. *n* = 10, ^∗^*P* < 0.05. (c) Mouse BALF samples were obtained as described in Figure 3(b). Flow cytometry analysis of BALF was performed at Body Fluid mode to obtain total counts of alveolar leukocytes. *n* = 10, ^∗^*P* < 0.05. (d) After centrifugation at 1,000 g, cell pellets from BALF were resuspended in serum and subjected to Wright-Giemsa staining on a glass slide. The counts of monocyte/macrophage (MN), neutrophil (PMN), and lymphocyte (Lym) and the ratio (%) of each cell type were obtained. *N* = 10.

One of the most prominent characteristics of ALI is the overwhelming infiltration of leukocytes into the alveoli [2, 3]. As expected, few leukocyte-containing alveoli were found in Sham and P5 (P5) mouse lungs (Figure 5(a)). At 20 hr post-ALI, extensive presence of leukocytes was found in over 50% alveoli (Figure 5(b), ALI). In the ALI+P5 group, however, the percentage of leukocyte containing alveoli was reduced by 59.8%, as compared with the ALI group (Figure 5(b)). These observations suggest that the protection conferred by P5 in ALI is due to its inhibitive effects on inflammation by reducing leukocyte infiltration into the alveolar cavity.

Flow cytometry analysis of BALF showed that the total number of alveolar leukocytes in each mouse was increased by nearly 5-fold from 0.85 × 10^5^ in sham mice to 5.83 × 10^6^ in ALI mice (Figure 5(c)). The administration of P5 in ALI mice reduced the leukocyte counts to 2.90 × 10^6^. Wright-Giemsa staining was performed to analyze the ratio of each cell type in BALF. The predominant cell type in Sham BALF was monocytes (93%, Figure 5(d)). The ratio of alveolar neutrophil was increased from 4.41% in sham to 86.52% in the ALI group. Thus, the near 50% reduction in leukocyte counts in ALI BALF caused by P5 was largely attributable to the suppression of neutrophil recruitment.

## 4. Discussion

### 4.1. P5 As a Broad Spectrum Cx HC Blocker

Cx isoforms are extensively and diversely expressed in virtually all tissue types [14–16, 22, 23]. The differences in cellular compartments connected by HCs and GJs endow these Cx channels with distinct functions. However, defining the distinctions has proven to be challenging due to the lack of selective approaches to interfere HCs or GJs. A most recent progress in developing HC interfering agent is the establishment of Cx43 peptide mimetic P5 [10]. Unlike its parental Cx43 mimetic GAP26, P5 does not affect Cx43 GJ formation and permeability, displaying its selectivity for HCs. Moreover, the HC blocking property of P5 is applicable under not only physiological conditions but also pathological conditions such as sepsis, ischemia [10], and ALI (this study), making it suitable for the investigation of the role of Cx HCs in various disease models.

Most tissues express multiple Cx isoforms [14–16, 22, 23]. Genetic manipulation of the expression of a Cx isoform may cause comprehensive expression of cohabitated Cxs [22]. Therefore, investigations of the functional significance of Cx HCs entail a simultaneous intervention of all Cx HCs while not compromising the formation of GJs. By demonstrating the capacity to reduce HC permeability in a wide range of cell types that express distinct sets of Cx isoforms, P5 may serve as a broad-spectrum blocker of Cx HCs.

The shared HC blocking property of P5 and its parental peptide GAP26 suggests that both peptides contain an HC blocking domain [10]. HepG2 cells express Cx26 and Cx32, whereas astrocytes express Cx26 and Cx30 besides Cx43. Both Cx26 and Cx30 contain a sequence in the EL1 overlapping the last 5 amino acids of P5 (NVCYD, Table 1). The EL1 of Cx32, on the other hand, contains a sequence of the last 4 amino acids of P5 (VCYD, Table 1). Interestingly, the VCYD sequence is also shared by GAP26 (**VCYD**KSFPISHVR) and P6 (**VCYD**KS), another 6-mer HC blocking peptide [10] indicating that this tetramer may be a critical attribute of these Cx HC peptide blockers. On the other hand, it is noteworthy that the levels of reduction in HC activity vary in Cx43-expressing macrophages (60%) in a previous study [10], HepG2 cells (28%), and Cx43-deficient astrocytes (13.5%), when P5 was applied at the same concentration, raising the possibility that HC blocking efficacy of P5 is, to some degree, Cx isoform selective. Analysis of the sensitivity of HCs formed by well-defined Cx isoform(s) to P5 may shed valuable insights on the mechanism underlying the HC blocking property of P5 and facilitate its application in various model systems.

### 4.2. Cx HCs Participate in the Pathogenesis of ALI

At least 7 Cx isoforms have been identified in alveolar and pulmonary vascular cells, among which Cx37, Cx40, and Cx43 are the most widely expressed [14, 16]. The involvement of Cx channels in ALI was first reported when Parthasarathi et al. investigated the role of Cx43 in calcium signaling between pulmonary vascular endothelial cells and thrombin-induced microvascular permeability [13]. It was found that Cx43 channels are necessary for calcium wave propagation between endothelial cells, as deficiency in Cx43 expression or channel blockade by Cx43 mimetic peptides GAP26 and GAP27 can abolish interendothelial calcium conduction [13]. More importantly, these treatments can also attenuate LPS- or thrombin-induced increase in microvascular permeability [13, 17, 19], suggesting that Cx43 channels mediate elevated pulmonary vascular permeability and consequently contribute to the pathogenesis in ALI. Similar to its role in interendothelial communication, Cx43 channels can also mediate calcium signaling between macrophages and epithelial cells in alveoli [18]. Instead of contributing to ALI pathology, however, Cx43-mediated macrophage-epithelium communication appears to counter LPS-induced ALI by reducing inflammation [18]. Besides Cx43, Cx37 and Cx40 have also been reported to play important roles in ALI [12, 20, 21]. While these studies demonstrated the significance of Cx channels in the ALI, the role of Cx HCs in ALI was not characterized, as experimental approaches were not GJ or HC selective. With the establishment of P5 as a broad spectrum Cx HC blocker, we were able to demonstrate the critical contribution of Cx HCs to the pathogenesis of ALI.

Massive leukocyte infiltration into alveoli is a fundamental characteristic of dysregulated inflammation in ALI [3–6]. It has been previously shown that Cx (Cx43 in particular) channels facilitate the mobility and recruitment of innate immune cells such as neutrophils and macrophages during ALI [15, 16]. The reduction in leukocyte containing alveoli subsequent to P5 administration suggests that Cx HC permeability is positively associated with the mobility of inflammatory cells. This conclusion appears to contradict with a recent study in which a presumptive Cx43 HC blocker (GAP19) showed an inhibitory effect on LPS-dependent neutrophil chemotaxis, thus suggesting that Cx43 HCs are negatively associated with neutrophil recruitment [27]. While the reason for this discrepancy is not clear, it is interesting to note that the effect of GAP19 on HC permeability in LPS-treated neutrophils was not examined in the study, and therefore, a correlation between Cx43 HC permeability and neutrophil chemotaxis remains to be established [27]. On the other hand, we have previously demonstrated that GAP19 can increase HC permeability in LPS-treated macrophages [10], contrary to its HC blocking property shown in several other cell types under physiological and ischemic conditions [28, 29]. If we speculate that GAP19 has a similar effect on HCs in LPS-treated neutrophils as in macrophages, it would further support the stimulatory role of Cx HCs in leukocyte (neutrophils and macrophages) recruitment in ALI.

The mechanism underlying the regulation of leukocyte recruitment by Cx HCs during ALI remains to be clarified. During ALI, LPS may increase the release of HMGB1 by innate immune cells such as macrophages and vascular endothelial cells, which results in an elevated level of HMGB1 in BALF ([10, 11] and this study). HMGB1 is known to serve two roles during inflammation: as a proinflammatory agent to further stimulate immune cells and as a chemoattractant to trigger chemotaxis [30]. Thus, the reduction of HMGB1 release by alveolar cells that resulted from the blockade of Cx HCs may lead to the decrease of HMGB1 in BALF, thus effectively removing the chemotaxis effect of HMGB1 and consequently reducing the infiltration of innate (e.g., neutrophils and macrophages) immune cells. Consistent with this notion, the decrease of HMGB1 in BALF (52.05%) is commensurate with reductions in the ratio of leukocyte-containing alveoli (59.8%) and in counts of neutrophils (50.10%) and monocytes (42.77%) in P5-treated ALI mice. On the other hand, blockade of Cx HCs may attenuate cell activation and cell mobility through attenuation of ATP release. In a cell migration assay, we did find that P5 reduced the mobility of LPS-challenged macrophages; however, the extent of reduction (10%, *P* < 0.001, data not shown) may not fully explain its impact on leukocyte recruitment in alveoli, indicating that the attenuation of leukocyte recruitment in alveoli by HC blockade may be largely due to the removal of the chemotaxis effect of HMGB1, as a result of the Cx HC blockade and subsequent inhibition of its extracellular release.

### 4.3. Selective Regulation of HMGB1 by Cx HCs

Extracellular HMGB1 plays a critical role in ALI elicited by infectious as well as noninfectious causes [5]. We have previously shown that HMGB1 release by macrophages is an active process and is under the control of HCs [10, 11]. The demonstration in the current study that Cx HC permeability is also related to HMGB1 release by endothelial cells indicates that Cx HCs are a common mechanism underlying the regulation of HMGB1 release in immune as well as nonimmune cells.

Inflammation is a complex process orchestrated by various cell types and cytokines/chemokines. Surprisingly, Cx HC-dependent regulation of cytokine release appears to be HMGB1 selective. For instance, blocking Cx43 HCs did not affect the extracellular level of nitric oxide and a vast majority of 64 other cytokines/chemokines in LPS-treated macrophages [10]. Moreover, P5 did not cause a significant change in the protein level and phosphorylation state of PKR ([22] authors' unpublished observations), a cytosolic protein kinase that is critical for cross cytoplasm translocation of HMGB1 [8, 31]. These observations imply that the control of HMGB1 release by Cx HCs occurs at the final stages of nuclei-extracellular migration of HMGB1. Together with the plasma membrane localization of HCs, these results raise the possibility of direct interaction between Cx HCs and HMGB1 or HMGB1 carriers.

## 5. Conclusions

In this study, we demonstrated that P5, a 6-mer Cx43 mimetic peptide, can serve as a pan-Cx HC blocker. The blockade of HCs by P5 resulted in the inhibition of HMGB1 release by human pulmonary vascular endothelial cells, decrease in extracellular HMGB1 in ALI, reduction of alveolar infiltration of innate immune cells, protection against LPS-induced lung injury, and increase in the survival rate of ALI mice.

These interesting results raise several questions for future studies. For instance, although P5 exerts a broad inhibitory effect on HCs in cells expressing distinct connexins, it is not clear whether it has similar dynamics on HCs formed by different Cxs. Clarification of this question would entail testing the effect of P5 in model systems expressing selective Cxs. In addition, it is not known whether the inhibition of HMGB1 release is through disruption of the Cx43-ATP-P2X7-HMGB1 axis, as we had previously proposed. Moreover, it remained to be determined which Cx isoform(s) is involved in the mediation of HMGB1 release and the pathogenesis of lung injury. These important questions should be addressed in future studies, which then will help to complete a picture detailing the roles and underlying mechanisms of Cx HCs.

Accumulating evidence suggests that both Cx and extracellular HMGB1 contribute to the pathogenesis in ALI, ischemic injury, and carcinogenesis/metastasis [5, 22, 23, 32, 33]. Clarification of the role of Cx HCs in the control of HMGB1 release may help advance the understanding of these pathological events. In addition, the establishment of a Cx HC-selective blocker may attenuate disease progression and thus provide a potential therapeutic option for these potentially devastating illnesses. The possibility that VCYD tetramer is the critical element of HC blocking property may assist more effective designs of Cx HC blocking agents.

## Figures and Tables

**Table 1:** Amino acid sequences in the EL1 of human Cxs corresponding to P5.

Human Cx isoforms	Amino acid sequence corresponding to P5
Cx43	**ENVCYD**
Cx33	**ENVCYD**
Cx45	**ENVCYD**
Cx46	**ENVCYD**
Cx50	**ENVCYD**
Cx26	K**NVCYD**
Cx30	K**NVCYD**
Cx30.3	P**NVCYD**
Cx31	T**NVCYD**
Cx37	T**NVCYD**
Cx40	Q**NVCYD**
Cx47	D**NVCYD**
Cx59	R**NVCYD**
Cx32	NS**VCYD**
Cx25	K**NVC**F**D**
Cx31.1	S**NVC**F**D**
Cx31.9	RQT**CYD**
Cx36	NQA**CY**D
Cx23	NLF**CY**N
Cx31.3	KAA**C**F**D**

## Data Availability

All software used in this study is commercially or publicly available. Database was not applicable. Raw data is available upon request, but public access cannot be made available under information safety policies of Huaihe Hospital.

## Abbreviations

ALI: Acute lung injury
ALT: Alanine aminotransferase
AST: Aspartate aminotransferase
BALF: Bronchoalveolar lavage fluid
Cx: Connexin
EL1: First extracellular loop
GJ: Gap junction
HC: Hemichannel
HMGB1: High mobility group box 1
HPMVEC: Human pulmonary microvascular endothelial cells
ip: Intraperitoneal
LDH: Lactate dehydrogenase
LY: Lucifer yellow
PF: Paraformaldehyde.
